# A monoallelic variant in *EYA1* is associated with Branchio‐Otic syndrome in a Malian family

**DOI:** 10.1002/mgg3.1995

**Published:** 2022-06-14

**Authors:** Abdoulaye Yalcouyé, Oumou Traoré, Salimata Diarra, Isabelle Schrauwen, Kevin Esoh, Magda Kamila Kadlubowska, Thashi Bharadwaj, Samuel Mawuli Adadey, Mohamed Kéita, Cheick O. Guinto, Suzanne M. Leal, Guida Landouré, Ambroise Wonkam

**Affiliations:** ^1^ Division of Human Genetics, Department of Medicine, Faculty of Health Sciences University of Cape Town Cape Town South Africa; ^2^ Faculté de Médecine et d'Odondostomatologie, USTTB Bamako Mali; ^3^ Neurogenetics Branch, National Institute of Neurological Disorders and Stroke Bethesda Maryland USA; ^4^ Center for Statistical Genetics, Gertrude H. Sergievsky Center, and Department of Neurology Columbia University Medical Center New York New York USA; ^5^ Service d'ORL, Centre Hospitalier Universitaire de Gabriel Touré Bamako Mali; ^6^ Service de Neurologie, Centre Hospitalier Universitaire du Point “G” Bamako Mali; ^7^ Taub Institute for Alzheimer's Disease and the Aging Brain, Columbia University Medical Center New York New York USA; ^8^ McKusick‐Nathans Institute and Department of Genetic Medicine Johns Hopkins University Baltimore Maryland USA

**Keywords:** Africa, Branchio‐otic syndrome, *EYA1*, Mali, syndromic hearing impairment

## Abstract

**Background:**

Branchio‐otic syndrome (BO) is one of the most common types of syndromic hearing impairment (HI) with an incidence of 1/40,000 globally. It is an autosomal dominant disorder typically characterized by the coexistence of branchial cysts or fistulae, malformations of the external, middle, and inner ears with preauricular pits or tags and a variable degree of HI. Most cases of BO have been reported in populations of European ancestry. To date, only few cases have been reported in people from African descent.

**Methods:**

After a careful clinical examination, a pure tone audiometry was performed. DNA was extracted from peripheral blood and whole exome, and Sanger sequencing were performed for genetic analysis.

**Results:**

Eight individuals from a large non‐consanguineous Malian family, with autosomal dominant inheritance were enrolled. The ages at diagnosis ranged from 8 to 54 years. A high phenotypic variability was noted among the affected individuals. Four patients presented with a post‐lingual and mixed type of HI, one individual had conductive HI while three had normal hearing but presented other BO features namely branchial fistulae and preauricular sinus. Serum creatinine level and renal ultrasonography were normal in three affected individuals who performed them. Genetic testing identified a monoallelic pathogenic variant in *EYA1* (c.1286A > G; p.Asp429Gly) segregating with BO syndrome in the family.

**Conclusion:**

This is the first genetically confirmed case of BO syndrome caused by *EYA1* variant in the sub‐Saharan African population, expanding the genetic spectrum of the condition.

## Graphical abstract

Most cases of Branchio‐otic syndrome (BO) have been reported in populations of European ancestry. Using Whole Exome, and Sanger Sequencing, we identified a monoallelic pathogenic variant in EYA1 [c.1286A>G; p.(Asp429Gly)] segregating with Branchio‐otic syndrome (BO)syndrome, in eight individuals from a large non‐consanguineous Malian family, with autosomal dominant inheritance. It is the first time the identified variant has been reported in Africa, expanding the genetic spectrum of the disease.

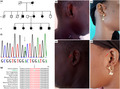

## 1 INTRODUCTION

Branchio‐oto‐renal syndrome (BO) is an autosomal dominant (AD) condition that is one of the most common forms of syndromic hearing impairments (SHI) with an estimated incidence of 1/40,000 people (Chen et al., 2019). It is a heterogeneous condition typically characterized by the coexistence of branchial cysts or fistulae, malformations of the external, middle, and inner ears with preauricular pits or tags, diverse degrees of HI and renal symptoms (OMIM# 113650). The syndrome is defined as Branchio‐Oto syndrome (BO) (OMIM# 602588) in the absence of renal abnormalities (Song et al., 2013). The clinical diagnosis is currently guided by the major and minor criteria of the Branchio‐oto‐renal spectrum disorder (BORSD) defined by Chang et al. (Chang et al., 2004). There are three genes (*EYA1*; OMIM# 601653; *SIX1*; OMIM# 601205, and *SIX5*; OMIM# 600963) that are known to cause BOR/BO syndrome. Mutations in *EYA1* are the most common causes of BOR/BO syndrome, and more than 200 pathogenic variants have been identified in various populations (Chen et al., 2019). Despite these numerous variants reported worldwide, none has previously been identified in the sub‐Saharan population with black ancestry. Here, we report the first confirmed case of BO syndrome caused by a heterozygous pathogenic missense variant in *EYA1* (c.1286A > G; p.Asp429Gly) in a large Malian family.

## 2 METHODS

### 2.1 Participants’ recruitment

All participants included in this study were recruited at the Department of Neurology of the Teaching Hospital of Point G, Bamako, Mali. After a record of the medical and family history followed by a pedigree description, all participants were carefully evaluated by medical geneticists (AW, GL) for the description of dysmorphological signs, and ENT specialists (MK) for an otological examination including otoscopy, Pure Tone Audiometry (PTA) for air and bone conduction. HI of common acquired causes was ruled out based on the medical history and otoscopy results. The degree of HI was classified according to the recommendation number 02/1 of the Bureau International d'Audiophonologie (BIAP), Belgium (www.biap.org). Moreover, kidney morphology and function were investigated through renal ultrasound and serum creatinine levels. The BORSD criteria were used for the clinical diagnosis (Chang et al., 2004).

### 2.2 Molecular analysis

Genomic DNA was extracted from peripheral blood using the QIAGEN Gentra Puregene Blood DNA Kit C (Germantown, MD), following the manufacturer protocol, in the laboratory of neurogenetics of the teaching hospital of Point G, Bamako, Mali.

### 2.3 Whole exome sequencing

**FIGURE 1:**
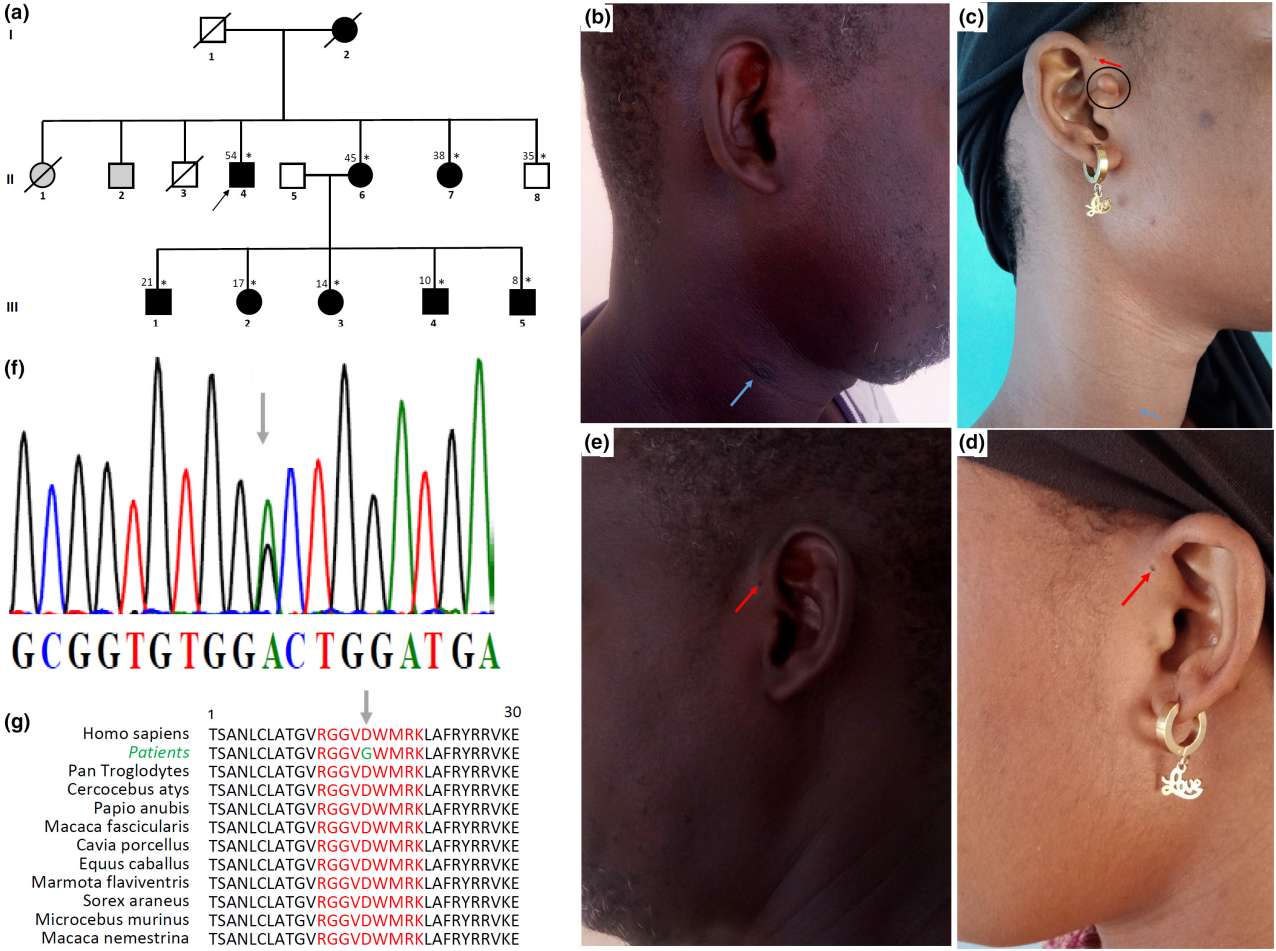
Phenotypic and genotypic features of the family with BO syndrome. (a) pedigree of the family showing an autosomal dominant pattern of inheritance, asterisks indicating those seen in clinic, the black arrow indicates the proband, and shaded individuals are reportedly affected but not seen in clinic, (b and e) images of the patient II.4, with the blue arrow indicating the right branchial fistula and the red arrow indicating preauricular sinus, (c and d) images of the patient III.2, with the red arrow indicating bilateral periauricular sinus, the black circle indicating the preauricular tag and the blue arrow showing the right branchial fistula, (f) chromatogram displaying the c.1286A > G variant indicated by the gray arrow, (g) the Asp429 residue conservation among different species indicated by gray arrow.

We initially sent DNA samples of individuals II.5 and II.6 (Figure 1a) for exome sequencing at Omega Bioservices (Norcross, GA, USA). The library preparation was performed with an Illumina Nextera Rapid Capture Exome Kit® (Illumina, San Diego, CA, USA) following the manufacturer's instructions, and the resulting libraries were hybridized with a 37‐Mb probe pool to enrich exome sequences (Wonkam‐Tingang et al., 2020). Sequencing was performed on an Illumina HiSeq 2500 sequencer using the pair‐end 150 bp run format. Sequence data were processed using the Illumina DRAGEN Germline Pipeline v3.2.8. Briefly, high‐quality reads were aligned to the human reference genome GRCh37/hg19 using the DRAGEN software version 05.021.408.3.4.12 and, after sorting and duplicate marking using Picard, variants were called, and individual genomic variant call format (gvcf) files were generated using the genome analysis toolkit (GATK) software v4.0.6.0 (McKenna et al., 2010). Joint single nucleotide variant (SNV) and Insertion/Deletion (Indel) variant calling were also performed using GATK. The sex of the two individuals undergoing exome sequencing were verified using plinkv1.9 (Chang et al., 2015). Familial relationships for these two family members were verified via Identity‐by‐Descent sharing (plinkv1.9) and the Kinship‐based INference for Gwas (KING) algorithm (Chang et al., 2015; Manichaikul et al., 2010).

### 2.4 Annotation and filtering strategy

ANNOVAR was used for variants filtering and annotation (https://annovar.openbioinformatics.org/) and custom scripts. Variants were first prioritized based on the inheritance model, considering an AD mode of inheritance. Subsequently, we considered rare variants with a minor allele frequency (MAF) <0.0005 in all populations of the genome aggregation database (gnomAD) were retained. Known pathogenic HI variants listed in ClinVar were also retained, regardless of their frequencies. dbNSFP v3.0 was used to evaluate missense variants, with 17 bioinformatic tools predicting the deleterious effects of the identified variants. We used several coding variants prediction tools including Sorting Intolerant from Tolerant (SIFT), polymorphism phenotyping v2 (PolyPhen‐2) × 2, Mutation Assessor, the likelihood ratio test (LRT), Mendelian clinically applicable pathogenicity (M‐CAP) score, Rare Exome Variant Ensemble Learner (REVEL), MutPred, protein variation effect analyzer (PROVEAN), MetaSVM, and MetaLR, while MutationTaster, Eigen, Eigen‐PC, functional analysis through Hidden Markov models (FATHMM‐MKL), combined annotation‐dependent depletion (CADD) score, and deleterious annotation of genetic variants using neural networks (DANN) score were used to annotate variants (Choi et al., 2012).

Adaptive boosting (ADA) and random forest (RF) scores derived from dbscSNV v1.1 were used to predict the deleterious effect of variants within splicing consensus regions (−3 to +8 at the 5′ splice site and −12 to +2 at the 3′ splice site). We used phyloP, Genomic Evolutionary Rate Profiling (GERP), SiPhy, and phastCons scores to estimate the evolutionary conservation of the nucleotides and amino acid residues at which the variants occurred (Pollard et al., 2010). The hereditary hearing loss homepage (HHL), online Mendelian inheritance in man (OMIM), human phenotype ontology (HPO), and ClinVar databases were used to determine if there were any existing associations between the identified variants and genes and HI. Candidate variants were considered when: (1) they occurred in known HI genes (and genes expressed in the inner ear); (2) they had a predicted effect on protein function or pre‐mRNA splicing (nonsense, missense, start‐loss, frameshift, splicing, start‐loss, etc.); and (3) they co‐segregated with the phenotype within the family (Wonkam‐Tingang et al., 2020).

### 2.5 Sanger sequencing

While exome sequencing was in process, Sanger sequencing was performed for all the available family members II.4, II.6, II.7, II.8, III.1, III.2, III.3, III.4, and III.5 (Figure 1a). Primers to target coding exons including splice sites of the *EYA1*, *SIX1*, and *SIX5* genes (available upon request) were validated using NCBI BLAST and ordered through Integrated DNA Technologies (IDT DNA, Coralville, IA, USA). The optimal annealing and extension temperatures for the PCR were 58°C and 70°C for 30 s and 40 s. PCR‐amplified DNA products were Sanger sequenced using a BigDye™ Terminator v3.1 Cycle Sequencing Kit and an ABI 3130XL Genetic Analyzer® (Applied Biosystems, Foster City, CA, USA) in the Division of Human Genetics, University of Cape Town, South Africa and the Neurogenetics Branch, NINDS, NIH (Bethesda, MD). Sequencing chromatograms were manually examined using FinchTV v1.4.0, and aligned in UGENE v34.0 to the *EYA1*, *SIX1*, and *SIX5* reference sequences (ENSG: ENST00000340726.8; ENST00000645694.3, and ENST00000317578.7; retrieved from Ensembl browser), respectively.

### 2.6 Evolutionary conservation of amino acids and secondary structure analysis

We performed a multiple sequence alignment (MSA) of human *EYA1* gene with non‐human similar proteins to provide more evidence on the evolutionary conservation of the amino acid residue at which our candidate missense variant occurred. A PSI‐BLAST search against the nonredundant protein database of EYA1 was performed. Non‐redundant, non‐synthetic EYA1 proteins from all the different species in the 500 BLAST hits were manually retrieved as FASTA files. The MSA was performed using CLUSTAL Omega v1.2.4 (Sievers et al., 2011) and the MSA file was visualized using Jalview v2.10.5 (Waterhouse et al., 2009). Furthermore, PSIPRED v4.0 (Buchan & Jones, 2019) and Swiss‐Model (Waterhouse et al., 2018) were used to assess the secondary structural features of both protein forms. Additionally, the InterPro database was queried via the InterProScan web service (Jones et al., 2014) to identify domains and potential domain changes for the protein.

### 2.7 Protein modeling

The *EYA1* homolog 1 isoform 1 (NM_001370335.1) protein sequence (NP_001357264.1) was retrieved from the NCBI GenePept database in FASTA format and secondary structures were predicted for the wild type as well as mutant sequences using PSIPRED workbench (http://bioinf.cs.ucl.ac.uk/psipred). The three‐dimensional (3D) structure of EYA1 G429 mutant protein was modeled using Modeler v10.1 based on the EYA1 wild type protein three‐dimensional structure that was retrieved from the AlphaFold protein structure database (https://alphafold.ebi.ac.uk/entry/Q99502). The three‐dimensional structure of the mutant protein was then refined using the Seok lab's Galaxy Refine algorithm (Heo et al., 2013). The PyMol software (Schrödinger, 2019) was used for hydrogen bond analysis and structure visualization, while the ExPASy Protparam web service (https://web.expasy.org/protparam/) was used to investigate the effect of the mutation on *EYA1* physicochemical properties.

## 3 CASE DESCRIPTION

### 3.1 Participants phenotypes

We enrolled eight affected individuals (four males and four females) and one unaffected from a single family. Affected family members presented with HI and other ear deformities with variable expression segregating in an autosomal dominant pattern (Figure 1a). The mean age at diagnosis was 25.8 years (ranging from 8 to 54 years). All the patients had at least one major criteria, fulfilling the BORSD clinical diagnosis criteria. Four of them presented with a mixed type of HI from mild to profound, one had a conductive HI, and three had a normal hearing. The onset of the HI was post‐lingual in five individuals (*n* = 5/8) and was symmetrical and asymmetrical in two and in three patients, respectively. A branchial fistula was observed in seven patients (87.5%) and absent in one patient (12.5%) (Figure 1b,c). It was bilateral in three patients (42.9%) (*n* = 3/7) and unilateral in four (57.1%) (*n* = 4/7). A preauricular sinus was seen in seven patients (87.5%) and was bilateral in six patients (Figure 1c,d). Only one patient (III.2) had a minor criterion, a preauricular tag in the right side (Figure 1c). Renal ultrasonography and creatinine levels were performed in three patients (II.4, II.6, and III.2), and were all normal. The clinical and laboratory findings are summarized in Table 1.

**TABLE 1:** Clinical and laboratory findings in the patients with BO syndrome

Patients	Age/sex	Physical signs	Creatinine level	Pure tone audiometry	Renal ultrasonography
*II.4*	54/M	Left preauricular sinus, right branchial fistula	Normal	Moderate symmetrical and mixed HI	Normal
*II.6*	45/F	Bilateral preauricular sinus and branchial fistula	Normal	Severe asymmetrical and mixed HI	Normal
*II.7*	38/F	Right branchial fistula	NP	Slight asymmetrical conductive HI	NP
*III.1*	21/M	Bilateral branchial fistula and preauricular sinus	NP	Normal	NP
*III.2*	17/F	Right branchial fistula, bilateral preauricular sinus, right ear tag	Normal	Moderate to severe asymmetrical mixed HI	Normal
*III.3*	14/F	Bilateral preauricular sinus	NP	Normal	NP
*III.4*	10/M	Bilateral branchial fistula and preauricular sinus	NP	Slight symmetrical mixed HI	NP
*III.5*	8/M	Bilateral branchial fistula and preauricular sinus	NP	Normal	NP

*Note*: Age in years.

Abbreviations: F, female; HI, hearing impairment; M, male; NP, not performed.

### 3.2 Sanger sequencing confirmation of variants

Sequencing of the *SIX1* and *SIX5* genes did not reveal any pathogenic variant. However, sequencing of the *EYA1* gene identified a heterozygous missense variant at position c.1286A > G (NM_000503.6), leading to the amino acid change p.Asp429Gly (Figure 1f). Sequencing of other family members showed that all affected individuals but not the unaffected family member (II.8) carried the variant.

### 3.3 Exome sequencing and confirmation of candidate gene variant

The average target region coverage was about 225X, with 96.30% of the target region being covered to a depth of 10 X or more. After applying our various filtering criteria described in the methods section, the candidate variant identified through Sanger sequencing was found (*EYA1*; OMIM# 601653, c.1286A > G (NM_000503.6), p.Asp429Gly). The variant was predicted to be damaging by most of the in silico tools, including MutationTaster, FATHMM‐MKL, Eigen‐PC, CADD, and DANN (Table S1). The variant was predicted to occur in conserved region of the genome and was absent from the gnomAD, UK10K, Greater Middle East (GME) variome project databases, as well as the SNP Database (dbSNP) (Table S2). Based on the American College of Medical Genetics' (ACMG) guidelines for the interpretation of sequence variants, the variant was classified as pathogenic (PM1, PS1, PM2, PP2, and PP3) (Oza et al., 2018; Richards et al., 2015).

### 3.4 Evolutionary conservation of amino acids

A PSI‐BLAST search of *EYA1* (NP_000494.2) against the nonredundant protein database found the Asp429 residue to be highly conserved across all non‐human species retrieved in the top 500 BLAST hits (Figure 1g). As expected, there was substantial conservation across an extensive amino acid block (on which the variant resides) which forms the thioredoxin/Genetic Diversity Statistics (GST)–C‐terminal binding domain. This was consistent with the GERP ++RS (5.44) and PhyloP (7.97) scores for conservation, indicating a strong evolutionary and functional constraint on the mutant position.

### 3.5 Protein modeling: Secondary structure analysis and domain search

**FIGURE 2:**
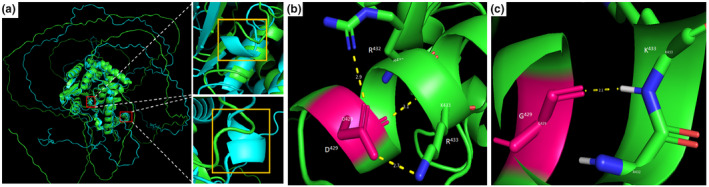
Three‐dimensional (3D) secondary analysis and hydrogen bond analysis of the EYA1 protein (NP_000494.2). (a) superimposed three‐dimensional structures of EYA1 wild type (green) and mutant (light blue) showing some regions with secondary structural changes (loss of helices in wild type or gain of helices in mutant), (b) wild type and (c) mutant protein structures. The wild type aspartate (D)‐429 residue forms three hydrogen bonds with arginine (R)‐432 and lysine (K)‐433. The mutant glycine (G)‐429 residue forms a single hydrogen bond with lysine (K)‐433.

Secondary structure analysis of wild type and mutant EYA1 sequences predicted multiple changes in the mutant protein due to the variant: this included loss of a helix structure at 107AA108, shortening of multiple helices (320DLER324 for instance), and gain or extension of helices and strands in the mutant (Figure S1a,b). These secondary structural changes were apparent in the three‐dimensional structures (Figure 2a). The three‐dimensional structure of wild type EYA1 indicates three hydrogen bonds formed between Aspartate‐429 (D^429^) and Arginine‐432 (R^432^) and Lysine (K^433^) (Figure 2b). In the mutant three‐dimensional protein, however, the mutant Glycine residue G^429^ forms a single hydrogen bond with K^433^ (Figure 2c). Based on the physicochemical properties, the mutant Glycine‐429 residue reduces the net charge of the mutant protein by −1, and consequently renders the protein more unstable by increasing its instability index.

## 4 DISCUSSION

To date, three genes (*EYA1*, *SIX1*, and *SIX5*) have been implicated in BO syndrome. The *EYA1* gene is the most common gene associated with BO, and accounts for ~40% of all cases. *EYA1* encodes for a protein that plays a role in regulating the activity of other genes. The EYA1 protein interacts with several other proteins, including a group known as SIX proteins, to turn on (activate) and turn off (inactivate) genes that are important for normal development. Eyes absent (EYA) is a transcriptional coactivator, and an aspartyl‐based protein tyrosine phosphatase that interacts with a broad variety of signaling pathways to regulate the development and homeostasis of organs and tissues such as eye, muscle, kidney, and ear. The variant (p.Asp429Gly) herein analyzed is predicted to alter the net charge of the EYA1, impart changes to the secondary structure of the protein, alter hydrogen bond formation, and reduce the stability of the protein. Moreover, the mutant Glycine429 residue which resides in the aspartyl‐based protein tyrosine phosphatase active site of the protein is smaller than the wild type Aspartate residue. Although Aspartate‐429 is not directly involved in the EYA1 phosphatase activity, it might be important in EYA1 interaction with its cofactors. Indeed, among its molecular functions, based on Gene Ontology, is metal ion binding, in which Mg^2+^ is identified as a cofactor. Therefore, the combined effects of net charge change, alteration of active site pocket, secondary structural changes, and instability are expected to affect the overall function of EYA1. The resulting genetic changes affect the development of organs and tissues before birth, which leads to the characteristic features of BOR/BO syndrome (Chang et al., 2004). Despite the high number of cases reported elsewhere, only three studies have been reported in Africa, of which two were genetically confirmed (Clarke et al., 2006; Mosrati et al., 2011; Nasir et al., 2018). Moreover, these two genetically confirmed cases are not of Black ancestry, a Tunisian and an Afrikaner. While the Afrikaner patient carried an *EYA1* variant, the BOR in the Tunisian patients was caused by a variant in *SIX1*. This is probably due to the lack of genetic testing facilities in many sub‐Saharan African countries or the attribution of most HI to environmental causes that do not necessitate further genetic investigation. The BOR/BO syndrome is a heterogeneous condition with high phenotype variability among individuals even in the same family as seen in our study. The phenotype found here is similar to what Namba et al. reported (Namba et al., 2001) and different from other studies such as the one from Clarke et al. (2006), confirming the heterogeneity of the disease. The mixed HI was the most common type of HI seen in this study, corroborating what was reported in patients with BOR/BO syndrome in the literature (Chen et al., 1995). In addition to this, the absence of renal morphological and functional abnormalities sustains the diagnosis of BO syndrome as reported in other studies (Mosrati et al., 2011; Namba et al., 2001). This condition is predominantly inherited in an AD manner (~90%) as observed in the family reported here. Hundreds of *EYA1* variants were reported in numerous families around the world. Interestingly, our report represents the third, worldwide, and the first in Africa of this variant (p.Asp429Gly). This variant was previously reported in a family from the United States with multiple affected individuals and in a sporadic case from Japan (Namba et al., 2001; Orten et al., 2008).

It would be interesting to further investigate the ancestry of the American family as most African Americans are of West African descent.

## 5 CONCLUSION

We identified a monoallelic variant in the *EYA1* gene in a Malian family with BO syndrome. It is the first time the identified variant has been reported in Africa, and the third time worldwide. With the decreasing cost of exome sequencing, genetic and genomic studies of the African population could identify more HI‐associated variants or genes which will improve our understanding of the pathophysiology of this condition.

## CONFLICT OF INTEREST

The authors declare that they have no conflict of interest.

## AUTHOR CONTRIBUTIONS

GL, SML and AW conceived the study, AY, OT, IS and SD developed the methodology. AY, SD, SMA, MK, COG performed clinical recruitment and molecular analysis. IS, KE, MKK, TB, analysed and interpreted the whole exome sequencing data; AY, OT wrote the first draft. AY, KE, AW, GL, IS, SML critically revised successive drafts of the manuscript. AW, SML, and GL supervised the project. All authors have read and agreed to the published version of the manuscript.

## ETHICAL COMPLIANCE

The study was performed according to the guidelines of the declaration of Helsinki. This study was approved by the institutional ethics committees/institutional review board (IRB) of the Faculty of Medicine and Dentistry of Bamako, Mali (N°2020/129/CE/FMOS/FAPH), the University of Cape Town, Cape Town, South Africa (HREC REF 691/2020) and Columbia University (IRB‐AAAS2343). Informed consent and assent for minor participants were obtained prior to their enrolment in this project, including the permission to publish photographs.

## Supporting information


Supinfo

Figure S1
