# Supplementary material for: A monoallelic variant in EYA1 is associated with Branchio‐Otic syndrome in a Malian family
Source: Mol Genet Genomic Med. 2022 Jun 14;10(7):e1995. doi: 10.1002/mgg3.1995 (PMC9266589; doi:10.1002/mgg3.1995)
Supplement: Supplementary file 1 — Supinfo [file MGG3-10-e1995-s001.docx]

**Supplementary materials**

**Figure S1:** Secondary structure analysis of the EYA1 wildtype and mutant protein

**Table S1:** Description of the variant with pathogenicity prediction tools

**Table S2:** Allele frequency in SNP database

**Online Resources**

| ANNOVAR | <https://annovar.openbioinformatics.org/> |
| --- | --- |
| Bureau international d’audiophonologie (BIAP) | <https://www.biap.org/en/recommandations/recommendations/tc-02-classification> |
| ClinVar | <https://www.ncbi.nlm.nih.gov/clinvar/> |
| dbNSFP (including dbscSNV) | <https://sites.google.com/site/jpopgen/dbNSFP> |
| dbSNP | <https://www.ncbi.nlm.nih.gov/snp/> |
| DRAGEN germline pipeline | <https://emea.illumina.com/products/by-type/informatics-products/basespace-sequence-hub/apps/edico-genome-inc-dragen-germline-pipeline.html> |
| Ensembl | <https://www.ensembl.org/index.html> |
| Gene ontology (GO) | <http://geneontology.org/> |
| Genome aggregation database (gnomAD) | <https://gnomad.broadinstitute.org/> |
| Genome analysis toolkit (GATK) | <https://gatk.broadinstitute.org/hc/en-us> |
| Hereditary hearing loss homepage (HHL) | <https://hereditaryhearingloss.org/> |
| Human phenotype ontology (HPO) | <https://hpo.jax.org/app/> |
| Human splice finder (HSF) | <https://hsf.genomnis.com/home> |
| InterProScan | <http://www.ebi.ac.uk/InterProScan/> |
| MODELLER | <http://www.salilab.org/modeller> |
| NCBI-BLAST | <https://blast.ncbi.nlm.nih.gov/Blast.cgi> |
| Online Mendelian inheritance in man (OMIM) | <https://omim.org/> |
| PDB | <https://www.wwpdb.org/> |
| PSIPRED | <http://bioinf.cs.ucl.ac.uk/psipred/> |
| PYMOL | <http://www.pymol.org/> |
| RefSeq | <https://www.ncbi.nlm.nih.gov/refseq/> |
| Swiss-Model | <https://swissmodel.expasy.org/> |
| Uniprot | <https://www.uniprot.org/uniprot/Q9NZA1> |
| UK10K | <https://www.uk10k.org/> |
| World Health Organisation | <https://www.who.int/news-room/fact-sheets/detail/deafness-and-hearing-loss> |

|  | **c.1286A>G, (p.Asp429Gly)** |
| --- | --- |
| Predicted effect | Missense |
| GERP | 5.44 |
| PhyloP | 7.969 |
| PhastCons | 1 |
| SiPhy | 15.666 |
| SIFT | 0.001 |
| Polyphen2 HDIV | 0.899 |
| Polyphen2 HVAR | 0.826 |
| MutationAssessor High | 2.975 |
| LRT | 0 |
| M-CAP13 | 0.259301 |
| REVEL | 0.939 |
| MutPred | 0.924 |
| PROVEAN | Damaging |
| MetaSVM | Damaging |
| MetaLR | Damaging |
| MutationTaster | 1 |
| Eigen | 0.836 |
| Eigen-PC | 0.803 |
| FATHMM-MKL | Damaging |
| CADD | 26.8 |
| DANN | 0.999 |
| ACMG classification | Likely pathogenic |

RefSeq transcript used NM_172059

**Table S1:** Description of the variant with pathogenicity prediction tools

| **Databases** | **Allele frequency**  **Table S2:** Allele frequency in SNP database |
| --- | --- |
| **gnomAD** | Absent |
| **dbSNP** | Absent |
| **UK10K** | Absent |
| **Variome database** | Absent |
