# Supplementary figures and images for: A monoallelic variant in EYA1 is associated with Branchio‐Otic syndrome in a Malian family
Source: Mol Genet Genomic Med. 2022 Jun 14;10(7):e1995. doi: 10.1002/mgg3.1995 (PMC9266589; doi:10.1002/mgg3.1995)

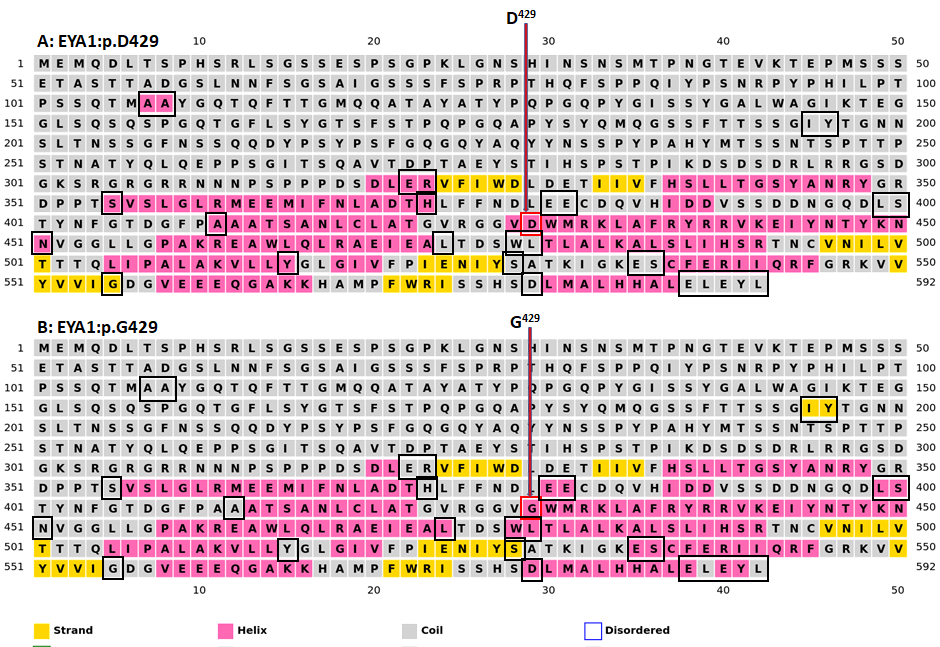

Supplement: Supplementary file 2 — Figure S1 [file MGG3-10-e1995-s002.tiff]
